# Osteopontin and Integrin Mediated Modulation of Post-Synapses in HIV Envelope Glycoprotein Exposed Hippocampal Neurons

**DOI:** 10.3390/brainsci10060346

**Published:** 2020-06-04

**Authors:** Farina J. Mahmud, Thomas Boucher, Shijun Liang, Amanda M. Brown

**Affiliations:** School of Medicine, Johns Hopkins University, Baltimore, MD 21287, USA; fmahmud1@jh.edu (F.J.M.); boucher152@gmail.com (T.B.); sjliang941231@gmail.com (S.L.)

**Keywords:** secreted phosphoprotein-1, human immunodeficiency virus type-1, synaptic plasticity, neuroprotection, neuroinflammation

## Abstract

The advent of Human Immunodeficiency Virus (HIV) antiretrovirals have reduced the severity of HIV related neurological comorbidities but they nevertheless remain prevalent. Synaptic degeneration due to the action of several viral factors released from infected brain myeloid and glia cells and inflammatory cytokines has been attributed to the manifestation of a range of cognitive and behavioral deficits. The contributions of specific pro-inflammatory factors and their interplay with viral factors in the setting of treatment and persistence are incompletely understood. Exposure of neurons to chemokine receptor-4(CXCR4)-tropic HIV-1 envelope glycoprotein (Env) can lead to post-synaptic degradation of dendritic spines. The contribution of members of the extracellular matrix (ECM) and specifically, of perineuronal nets (PNN) toward synaptic degeneration, is not fully known, even though these structures are found to be disrupted in post-mortem HIV-infected brains. Osteopontin (Opn, gene name *SPP1*), a cytokine-like protein, is found in abundance in the HIV-infected brain. In this study, we investigated the role of Opn and its ECM integrin receptors, *β*1- and *β*3 integrin in modifying neuronal synaptic sculpting. We found that in hippocampal neurons incubated with HIV-1 Env protein and recombinant Opn, post-synaptic-95 (PSD-95) puncta were significantly increased and distributed to dendritic spines when compared to Env-only treated neurons. This effect was mediated through *β*3 integrin, as silencing of this receptor abrogated the increase in post-synaptic spines. Silencing of *β*1 integrin, however, did not block the increase of post-synaptic spines in hippocampal cultures treated with Opn. However, a decrease in the PNN to *β*III-tubulin ratio was found, indicating an increased capacity to support spine growth. From these results, we conclude that one of the mechanisms by which Opn counters the damaging impact of the HIV Env protein on hippocampal post-synaptic plasticity is through complex interactions between Opn and components of the ECM which activate downstream protective signaling pathways that help maintain the potential for effective post-synaptic plasticity.

## 1. Introduction

Neurocognitive problems due to human immunodeficiency virus type 1 (HIV-1) infection are prevalent and comprise a spectrum of impairments that cannot be fully controlled by suppressive antiretrovirals. The virus enters the brain early and is less receptive to current therapy [1]. The advent of antiretrovirals has reduced the severity of neuronal degeneration in HIV infection, but it still affects 15–55% of the HIV+ population [1]. Understanding the detailed molecular mechanisms that instigate neurological impairments due to HIV infection is required to develop therapeutic approaches targeting this comorbidity.

Viral secreted factors and the inflammatory milieu elicited by HIV infection of peripheral and central immune cells are implicated in the neuronal injury observed in different experimental paradigms [2,3,4,5]. HIV viral proteins such as the non-structural protein Tat and Env glycoprotein (gp120) alter neuronal function and morphology by stimulating excitatory neuronal receptor NMDAR, which increases influx of Ca^2+^ ions and contributes to hyper-excitation [5,6,7,8,9]. Additionally, HIV-1 gp120 engages the chemokine receptors CXCR4 (X4) or CCR5 (R5) present on the surface of neurons to provoke neuronal hyper-excitability [7,10]. Interestingly, the X4-tropic gp120 (HIV-1 IIIB strain) can instigate the aberrant activation of CXCR4 and induce synaptodendritic damage [7,10,11,12].

In this context, we are interested in gaining more insight on the molecular mechanisms by which viral factors and the inflammatory milieu perturb synaptic function. Osteopontin (Opn), or secreted phosphoprotein 1 (*SPP1*), is a cytokine-like glycophosphoprotein found elevated in brain samples collected post-mortem from HIV infected subjects with neurocognitive impairments or encephalitis [13,14,15]. Plasma levels of Opn were correlated and indicative of HIV-related neurological abnormalities and immune activation in the brain [15]. Opn is secreted by myeloid derived cells such as infiltrating macrophages and resident microglia during neuroinflammation [15,16]. Peripherally, Opn participates in cell adhesion, chemotaxis, and pro-survival pathways [15,17,18,19]. In the central nervous system (CNS), Opn is elevated in neuroinflammatory conditions, attracts glia to damaged sites, act as a potential biomarker for microglial activation in response to ischemic injury and autoimmune disease [17,20,21]. In a subset of motor neurons, Opn was reported to play a crucial role in the neurodegeneration of ALS-resistant motor neurons by modulating the expression of matrix metalloproteinase-9 (MMP9) [22].

The important contribution of extracellular matrix (ECM)-integrin interactions to the fidelity of synaptic plasticity has been studied [23,24,25,26,27]. These insights should prove helpful in gaining a mechanistic understanding of the ECM-integrin ligand, Opn, and its role in HIV neuropathogenesis. Neurons harbor at least three of Opn’s receptors: *β*1 integrin, *β*3 integrin, and cluster of differentiation 44 (CD44) [19,28,29]. These receptors are cell adhesion molecules and are known to modulate and maintain synaptic plasticity in neurons [30,31,32,33,34]. Integrin-ECM interactions trigger outside-in signaling that result in actin remodeling in many type of cells [34]. Impaired integrin activation can negatively affect synaptic plasticity leading to behavioral deficits [34,35]. *β*1 integrin pathway activation by the proteolytic enzyme MMP9 has been shown to induce spine growth and degradation of a component of the ECM called perineuronal nets (PNN) [24,36]. Most recently PNN were shown to be degraded in the HIV-infected brain and attributed to an increased metalloproteinase (MMP) activity [23]. Due to their role in synaptic remodeling we hypothesized that excess Opn, secreted during HIV infection, engages these receptors and modulates synaptic machinery.

To model HIV-neuronal interactions in vitro, we studied Opn’s role on synaptic remodeling in the presence of HIV-1 Env protein from the IIIB strain on primary rat hippocampal neurons. Exogenous application of rat recombinant Opn at a concentration found in circulating serum of patients with various pathologies (100 ng/mL) were utilized to mimic high levels of secreted Opn [15,37,38]. Importantly, hippocampal neurons play a central role in learning and memory and understanding the molecular basis for their dysregulation in HIV-associated infection is critical. Moreover, we hypothesized that Opn acts as an upstream mediator that activates integrin function leading to altered synaptic integrity by influencing post-synaptic changes. In this regard, Opn might have an impact on synaptic density in the presence of dysregulated Ca^2+^ initiated by HIV-1 Env, since calcium signaling is pertinent in controlling plasticity related genes [39,40].

Compared to controls, we found alterations in hippocampal post-synaptic machinery after exposure to HIV-1 Env, as the density of dendritic spines containing the post-synaptic scaffolding protein PSD-95 was significantly decreased. This effect was reversed by co-treatment with recombinant Opn. To understand the mechanism of how Opn reverses the decrease in post-synapses in the presence of HIV-Env, *β*1- and *β*3 integrins were silenced. Surprisingly, silencing *β*1 integrin in the presence of Opn and HIV-1 Env further increased the number of PSD-95 post-synapses. This finding suggests that Opn and *β*1 integrin functioning in this context are not co-dependent. Likely due to their tight association with the ECM, silencing *β*1 integrins resulted in decreased expression of perineuronal nets (PNN) and increased growth of PSD-95 post-synaptic structures. Treatment with Opn alone also led to a decrease in the density of PNNs. In contrast, silencing *β*3 integrins did not decrease the number post-synapses in the presence of both Opn and HIV-1 Env, but interestingly it did in Opn-only treated neurons. This finding suggests the possibility of convergent signaling between *β*3 integrin and Env receptor pathways. Surprisingly, in HIV-1 Env only treated neurons, silencing *β*3 integrins also decreased the number of post-synapses suggesting activation of protective compensatory signaling that ultimately serves to block further damage.

## 2. Materials and Methods

### 2.1. Primary Hippocampal Neuron Culture

Dissociated primary hippocampal neurons were cultured from rat hippocampi digested with 1 mg/mL papain from E18 embryos (Neuromics) and depending on the downstream experiment, 20–40,000 cells were cultured on poly-D-lysine (PDL) coated coverslips (Neuvitro) for microscopy. The cells were supplemented with Neurobasal plus media containing Glutamax and B27+. CultureOne supplement was used to limit glial growth (Gibco), which according to manufacturer’s instructions, eliminates any glial growth when supplemented at DIV 0. Cells were grown at 37 °C and in 5% CO_2_ for 7 or 14 days in vitro.

### 2.2. SiRNA Silencing and Treatments

Receptor expression was modulated using anti-integrin silencing performed with the SiGENOME SMARTpool siRNA directed against rat *β*1 and *β*3 integrins using Dharmafect 1 transfection reagent on DIV 7 neurons. GFP-SiRNA were utilized to determine the ideal concentration of the transfection reagent and confirm successful transfection. Green fluorescent protein (GFP) positive puncta were observed in the entire well along with a low-level of cell death as observed by neurite blebbing.

DIV 12 neurons were subjected to HIV-1 Env (400 pM) and rat recombinant Opn (R&D systems, 100 ng/mL) for 48 h. Cells were harvested on DIV 14 for synaptic analysis and subjected to 4% paraformaldehyde fixation and subsequent antibody staining for immunofluorescence.

### 2.3. Immunofluorescence

Fixed cells were permeabilized with 0.2% Triton-X for 5 min followed by blocking with 10% goat serum for 1 h. Cells were probed with antibodies against post-synaptic protein PSD-95 (1:600, Neuromics MO50000), ***β***III-tubulin (Millipore MAB1637 1:100) and biotin conjugated wisteria floribunda lectin (WFA) which binds to the glycoproteins in PNN [41] (1:50, Sigma-Aldrich, L1516-2MG) at 4 °C overnight. Secondary antibodies conjugated with fluorophores (mouse AlexaFluor-488 Invitrogen A11017, rabbit AlexaFluor-568 Invitrogen A11011, 1:500 and 1:500 streptavidin AlexaFluor-568 Invitrogen S11226) were incubated for 1 h at room temperature after which the coverslips were mounted with ProLong Gold Antifade mountant with DAPI (Thermofisher P36941) and imaged. Images were acquired with Zeiss LSM 800 or Apotome one at 20× or 40× magnification. 22 slices of 6 µM Z-stack sections with a 0.28 µM interval were acquired and maximum intensity projection was applied.

### 2.4. Image Analysis

Confocal and apotome images (at least 12 per treatment) were quantified for the number of dendritic spines per neurons and the immunoreactivity of the markers used. All image analysis and quantification was done using custom codes on Matlab R2017a (MathWorks) available upon request.

### 2.5. Experimental Groups

The first portion of this study had four non-transfected groups. Vehicle + Opn- only received neurobasal plus media on DIV 12. Vehicle + Opn+ received neurobasal plus media with 100 ng/mL rat recombinant Opn. Env + Opn- were treated with neurobasal plus containing 400 pM of HIV-1 Env. Env + Opn+ neurons were co-treated with 100 ng/mL rat recombinant Opn and 400 pM of HIV-1 Env in neurobasal plus media.

Silencing studies were performed next with scrambled siRNA as controls, *β*1ITG siRNA to silence *β*1 integrins and *β*3ITG to silence *β*3 integrins. Silencing were performed on vehicle treated, Env + Opn+, Opn only, and Env only treated neurons. Scrambled controls were used for comparisons analysis with both *β*1 and *β*3 integrins.

### 2.6. Statistics

**Figure 1:**
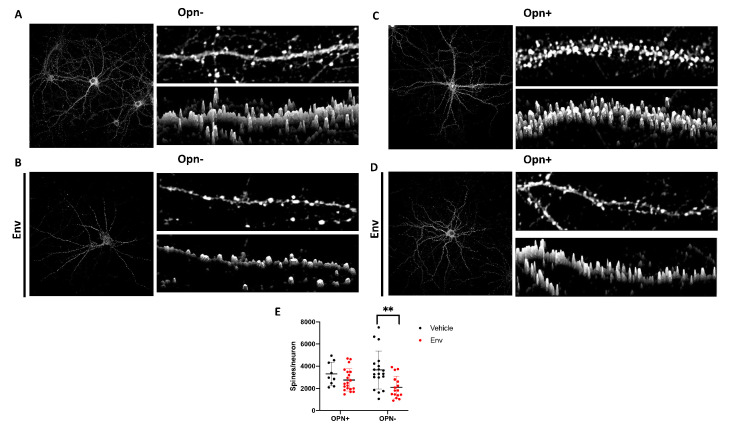
Opn counteracts HIV-1 Env mediated post-synaptic damage. Representative images of primary hippocampal neurons treated with vehicle (**A**), Env only (**B**), Opn only (**C**), and Env + Opn (**D**) are shown. Left panel shows image of the entire neuron and on its top-right is a section of the dendrite and 3D construction of the dendrite section below it. (**E**) shows grouped interleaved scatter graph for the number of synapses per neuron due to the four different treatments. The mean ± standard deviation (SD) are shown with the following number of sampling for each groups: *n* = 4 and 45 neurons for vehicle treated neurons, *n* = 3 and 34 neurons for Env only, *n* = 3 and 13 neurons for Opn only and *n* = 4 and 52 neurons for Env + Opn. Two-way ANOVA with Tukey’s post-test was performed to reveal statistical difference between groups (** *p* < 0.001).

**Figure 2:**
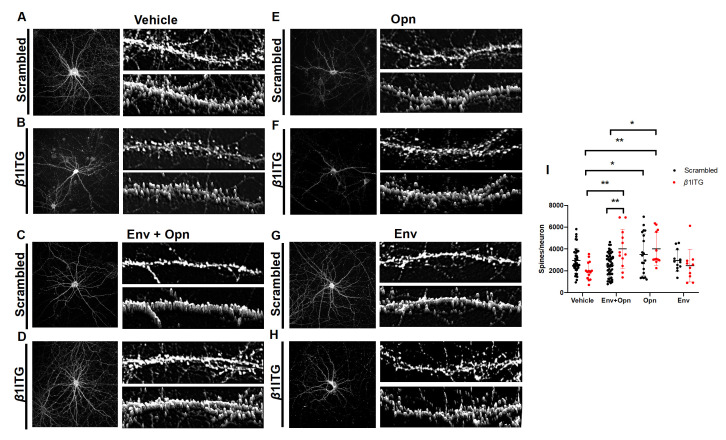
*β*1 integrin modulates spine density in the presence of HIV-1 Env and Opn. Representative images of primary hippocampal neurons transfected with either scrambled siRNA (**A**,**C**,**E**,**G**) or *β*1 integrin siRNA (**B**,**D**,**F**,**H**) are shown. Exogenous treatments include the following: Vehicle (**A**,**B**), Env + Opn (**C**,**D**), Opn only (**E**,**F**), and Env only (**G**,**H**). Left panel shows image of the entire neuron and on its top-right is a section of the dendrite and 3D construction of the dendrite section below it. (**I**) shows grouped interleaved scatter graph for the number of synapses per neuron due to the different treatments. The mean ± SD are shown with the following number of sampling for each groups: *n* = 5 and 117 neurons for vehicle treated scrambled transfected, *n* = 2 and 42 neurons for vehicle treated *β*1 integrin SiRNA transfected, *n* = 7 and 120 neurons for Env + Opn treated and scrambled transfected, *n* = 2 and 13 neurons for Env + Opn treated *β*1 integrin siRNA transfected, *n* = 2 and 66 neurons for Opn treated scrambled transfected, *n* = 3 and 28 neurons for Opn treated *β*1 integrin siRNA transfected, *n* = 2 and 16 neurons for Env only treated and scrambled transfected and *n* = 2 and 13 neurons for Env only treated *β*1 integrin siRNA transfected neurons. Two-way ANOVA with Tukey’s post-test was performed to reveal statistical difference between groups (* *p* < 0.01 and ** *p* < 0.001).

**Figure 3:**
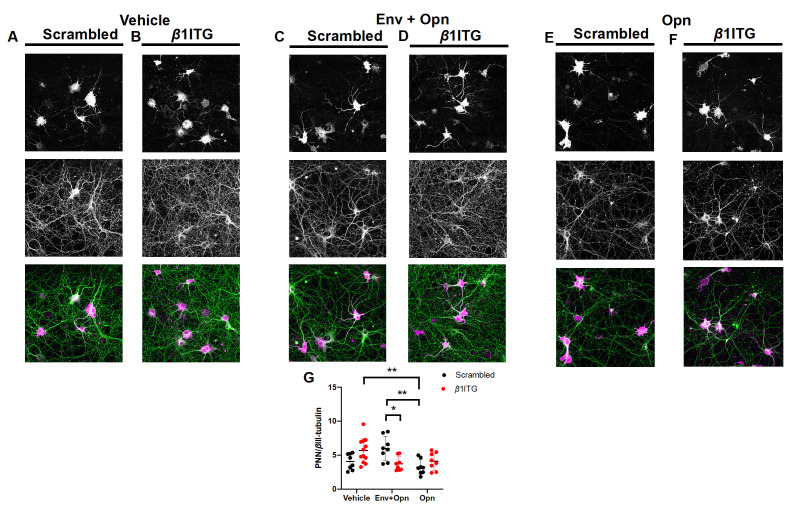
Perineuronal net (PNN) expression is decreased in *β*1 integrin silenced neurons in the presence of HIV Env and Opn. Representative images of hippocampal neurons stained with PNN glycoprotein marker (WFA) and neurite marker *β*III-tubulin are shown. Top panel shows PNN stain, middle shows *β*III-tubulin and bottom panel shows the merged image. Exogenous treatments include the following: Vehicle (**A**,**B**), Env + Opn (**C**,**D**) and Opn only (**E**,**F**). The neurons were transfected with either scrambled siRNA (**A**,**C**,**E**) or *β*1 integrin siRNA (**B**,**D**,**F**). The mean ± SD are shown and sampling was *n* = 2 for all groups. The quantified expressions of PNN/*β*III-tubulin are shown in (**G**). Two-way ANOVA with Tukey’s post-test was performed to reveal statistical difference between groups (* *p* < 0.01 and ** *p* < 0.001).

**Figure 4:**
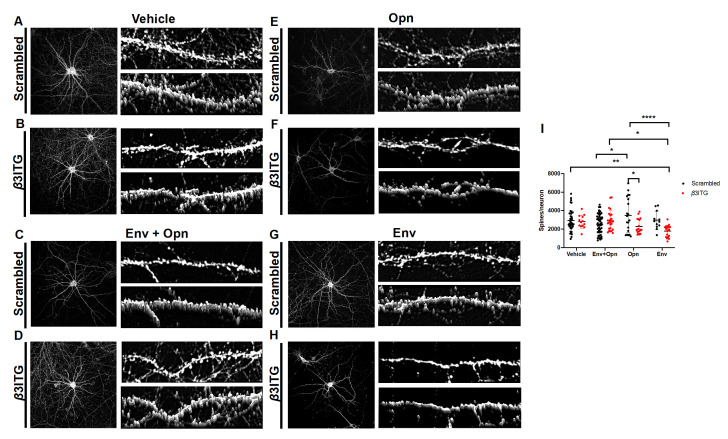
Opn acts via *β*3 integrins to modulate spine density. Representative images of primary hippocampal neurons transfected with either scrambled siRNA (**A**,**C**,**E**,**G**) or *β*3 integrin siRNA (**B**,**D**,**F**,**H**) are shown. Exogenous treatments include the following: Vehicle (**A**,**B**), Env + Opn (**C**,**D**), Opn only (**E**,**F**) and Env only (**G**,**H**). Left panel shows image of the entire neuron and on its top-right is a section of the dendrite and 3D construction of the dendrite section below it. (**I**) shows grouped interleaved scatter graph for the number of synapses per neuron due to the different treatments. The mean ± SD are shown with the following number of sampling for each groups: *n* = 5 and 117 neurons for vehicle treated scrambled transfected, *n* = 3 and 43 neurons for vehicle treated *β*3 integrin SiRNA transfected, *n* = 7 and 120 neurons for Env + Opn treated and scrambled transfected, *n* = 5 and 70 neurons for Env + Opn treated *β*3 integrin siRNA transfected, *n* = 2 and 66 neurons for Opn treated scrambled transfected, *n* = 3 and 73 neurons for Opn treated *β*3 integrin siRNA transfected, *n* = 2 and 16 neurons for Env only treated and scrambled transfected and *n* = 3 and 26 neurons for Env only treated *β*3 integrin siRNA transfected neurons. Two-way ANOVA with Tukey’s post-test was performed to reveal statistical difference between groups (* *p* < 0.01, ** *p* < 0.001, and **** *p* < 0.0001).

**Figure 5:**
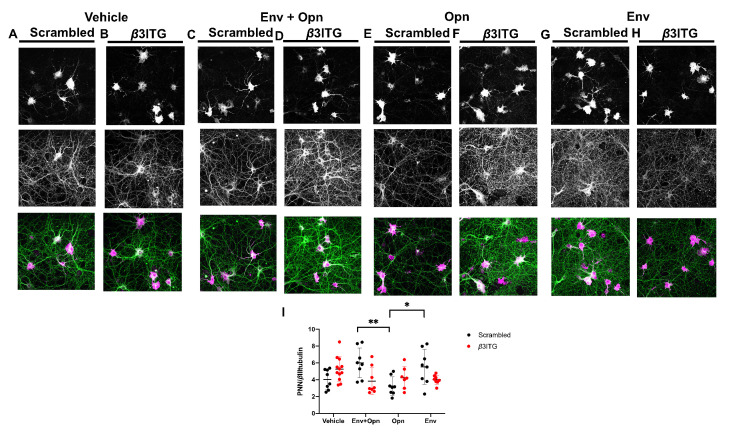
*β*3 integrins do not modulate PNN expression in the presence of HIV-1 Env and Opn. Representative images of hippocampal neurons stained with PNN glycoprotein marker (WFA) and neurite marker *β*III-tubulin are shown. Top panel shows PNN stain, middle shows *β*III-tubulin, and bottom panel shows the merged image. Exogenous treatments include the following: Vehicle (**A**,**B**), Env + Opn (**C**,**D**), Opn only (**E**,**F**), and Env only (**G**,**H**). The neurons were transfected with either scrambled siRNA (**A**,**C**,**E**,**G**) or *β*3 integrin siRNA (**B**,**D**,**F**,**H**). The mean ± SD are shown with and sampling was *n* = 2 for all groups. The quantified expressions of PNN/ *β*III-tubulin are shown in (**I**). Two-way ANOVA with Tukey’s post-test was performed to reveal statistical difference between groups (* *p* < 0.01 and ** *p* < 0.001).

All graphs are represented as mean ± standard deviation (SD). Statistics were done using Graphpad Prism 7. Two-way analysis of variance (ANOVA) with Tukey post hoc analysis were performed on multiple comparisons. Column factor (Degrees of freedom DF = 1) for Figure 1 was Env treated or not and row factor (DF = 1) was Opn treated or not. Column factor for Figure 2, Figure 3, Figure 4 and Figure 5 (DF = 1) where SiRNA transfection and row factors (DF = 3) were Opn only, Env only, Env + Opn, and vehicle treatments (exogenous treatments). Sampling (n) was determined by the number of times the experiments were repeated, the number of observations is the number of images acquired (individual point on the interleaved scatters), the number of neurons were the number of neurons analyzed, and the alpha value for significance was set to be 0.05.

## 3. Results

### 3.1. HIV-1 Env Mediates Post-Synaptic Remodeling by Decreasing the Number of Dendritic Spines, but Co-Treatment with Opn Reverses This Damage

The density and architectural integrity of post-synaptic dendritic spines has been implicated as molecular correlates of long-term potentiation. The number of dendritic spines reflect the status of neuronal plasticity as they protrude out of the dendrites, serving as inputs for pre-synaptic boutons extending from axons [42]. Thus, a higher number of dendritic spines indicates an increased number of contacts are being formed with the pre-synapse, and reflect the degree of synaptic strength. Therefore, we evaluated the number of dendritic spines formed by the post-synaptic scaffolding protein PSD-95 in the presence of Opn and HIV-1 Env. Two-way ANOVA analysis revealed a significant difference in post-synapses between vehicle control (Figure 1, black circles) and exposure of neurons to HIV-1 Env (Figure 1, red circles) (Column factor *p* = 0.0023) (Figure 1D,E). Exogenous application of HIV-1 Env only (Env+) induced a decrease in the number of dendritic spines per neuron when compared to vehicle-treated neurons (*p* = 0.0029) (Figure 1E). Three-dimensional image stack reconstruction, analyses also showed PSD-95 concentration preferentially on dendrites as opposed to localization to spines in neuronal cultures exposed to HIV-1 Env (Figure 1A,B). In contrast, on vehicle treated neurons PSD-95 post-synapses were predominantly distributed in spines. Co-treatment of neurons with HIV-1 Env and Opn (Env + Opn+) showed reversal of these damages as there were no differences between the vehicle+Env- or Opn only treated neurons (Figure 1A–D, 1E: left pair under OPN+). Co-treatment with Opn also distributed PSD-95 to the spines (Figure 1D).

### 3.2. Opn Acts Independently of Extracellular Matrix (ECM) Component, β1 Integrin, to Regulate Hippocampal Post-Synapses in the Presence of HIV-1 Env

To understand the mechanism utilized by Opn to achieve the reversal of post-synaptic damage inflicted by HIV-Env, one of its receptors, *β*1 integrin, was silenced (siRNA-KO). Two-way ANOVA revealed an interaction between silencing of *β*1 integrin and the exogenous treatments (Interaction *p* = 0.0003). This finding therefore, suggested that changes in spine density were contingent on both *β*1 integrin silencing and treatments with HIV-1 Env and Opn. Spine density was also found to be dependent on HIV-1 Env and Opn treatments (Row factor *p* = 0.0003). In this regard, there were no differences between *β*1 integrin silencing on vehicle-treated and scrambled transfected control neurons suggesting that this integrin does not regulate post-synaptic spine density during physiological conditions in DIV 14 neurons (Figure 2A,B,I:Vehicle circle pairs). Interestingly, *β*1 integrin silencing increased the number of post-synapses in neurons co-treated with Env + Opn+ (*p* = 0.0093) when compared to scrambled transfected (Figure 2C,D,I:Env + Opn circle pairs). This increase was also significant when compared with vehicle-treated neurons transfected with reduced *β*1 integrin expression (*p* = 0.0026) (Figure 2B,D,I:Vehicle, red circles vs Env + Opn, red circles). There were no differences between scrambled siRNA transfected, Opn-only treated neurons and *β*1 integrin siRNA-KO neurons (Figure 2I: Opn (circle pairs). These finding suggests that Opn’s ability to modulate spine PSD-95 post-synapses in hippocampal neurons is not dependent on *β*1 integrin. Additionally, no differences were found between scrambled siRNA transfected Env-only treated neurons and *β*1 integrin silenced neurons (Figure 2G,H,I: Env circle pairs).

### 3.3. Perineuronal Net (PNN) Expression Is Decreased in β1 Integrin Silenced Neurons Co-Treated with HIV-1 Env and Opn, and in Neurons Treated Only with Opn Compared to Vehicle Controls

Due to *β*1 integrin’s tight association with PNNs, we next wanted to understand how silencing of this integrin impacted the expression of PNNs, another key member of the ECM. We first quantified total PNN density on hippocampal neurons to determine whether the experimental treatments alone altered its expression. PNN expression was normalized to *β*III-tubulin levels (PNN/*β*3-tubulin) and two-way ANOVA revealed a significant interaction between exogenous treatments and siRNA transfections (Interaction *p* = 0.006) (Figure 3G). No differences were found between vehicle-treated neurons either transfected with scrambled siRNA, or *β*1 integrin silencing confirming that *β*1 integrins do not regulate the expression of PNN/*β*III-tubulin during physiological conditions in DIV 14 neurons (Figure 3A,B,G:Vehicle circle pairs). However, in the co-presence of HIV-1 Env and Opn there was a significant decrease in the PNN/*β*III-tubulin expression when compared to HIV-1 Env and Opn co-treated and scrambled siRNA transfected neurons (*p* = 0.0320) (Figure 3C,D,G: Env + Opn circle pairs). A decrease in PNN/*β*III-tubulin expression in Opn-only treated neurons was observed when compared to scrambled siRNA transfected HIV-1 Env and Opn co-treated neurons (*p* = 0.0049) (Figure 3C,E,G: Env + Opn, black circles vs Opn, red circles), and to vehicle-treated neurons transfected with *β*1 integrin silencing (*p* = 0.0070) (Figure 3B,E,G: Vehicle, red circles vs Opn, red circles). The decrease in the PNN/*β*III-tubulin ratio was not *β*1 integrin dependent since silencing this receptor in Opn-only treated neurons did not change the expression of PNN/*β*III-tubulin when compared to the scrambled transfected Opn-only treated neurons (Figure 3E–G: Opn, black vs red circles). Collectively, these results suggest that Opn treatment can override the damage signal induced by HIV Env to reduce the PNN/*β*III-tubulin ratio, and does so more effectively when *β*1 integrin expression is blocked (Figure 3G: Env + Opn, circle pairs).

### 3.4. β3 Integrins Are Required for Opn Induced Upregulation of Post-Synaptic Dendritic Spines

Next, *β*3 integrins were silenced to determine whether they were involved in the Opn mediated regulation of post-synapse phenotype. A significant interaction between siRNA transfections and the exogenous treatments was detected (Interaction *p* = 0.0010) (Figure 4I). Changes to spine density were also dependent on siRNA transfections (Column factor *p* = 0.0030). There were no significant differences between scrambled siRNA transfected and *β*3 integrin silencing siRNA control treated neurons, suggesting that these integrins do not regulate post-synaptic spines during physiological conditions (Figure 4A,B,I: Vehicle, circle pairs). There was however a decrease in the number of spines in Opn-only treated *β*3 integrin silenced neurons when compared to Opn-only treated scrambled siRNA transfected neurons (*p* = 0.0232) (Figure 4E,F,I: Opn, circle pairs). This finding suggests that Opn interacts with *β*3 integrins to regulate the density of spines in hippocampal neurons. Surprisingly, there was a significant decrease in the number of spines in HIV-1 Env treated neurons when *β*3 integrins were silenced compared to vehicle treated scrambled transfected neurons (*p* = 0.0063) (Figure 4I, Env, circle pairs), Env + Opn treated, *β*3 integrin silenced neurons (*p* = 0.0253) (Figure 4I, Env + Opn, red circles), and Opn-only treated scrambled transfected neurons (*p* < 0.0001) (Figure 4D,E,H,I, Opn, black circles).

### 3.5. In HIV-1 Env and Opn Treated Neurons, PNN Expression Is not Regulated by β3 Integrins

Next, we tested whether *β*3 integrins regulate the expression of PNNs in the presence HIV-1 Env and Opn. There was a significant interaction between siRNA transfections and exogenous treatments as revealed by two-way ANOVA (*p* = 0.0045) (Figure 5I). PNN expression was also highly dependent on the exogenous treatments (Row factor *p* = 0.0356). There were no differences between vehicle scrambled transfected neurons or their *β*3 integrin silenced counterparts suggesting that this receptor is not involved in regulating the PNN/*β*tubulin expression (Figure 5I: all groups and their circle pairs). There was a decrease in Opn only treated scrambled transfected neurons when compared to Env and Opn co-treated and scrambled transfected (*p* = 0.0071) and Env only treated and scrambled transfected (*p* = 0.0466) (Figure 5I: Env, black circles).

## 4. Discussion

The molecular and electrical properties of neuronal circuitry are governed by synapses. Indeed, synaptic integrity is crucial to maintain healthy communication between neurons and any interruption will trigger aberrant activity. Synaptic activity is governed by many complex factors contributed by neurons and glia, and any changes in the neuronal microenvironment will have a positive or negative impact [43]. Indeed, neuronal dysfunction during HIV infection in the form of morphological and functional changes that lead to a disruption the neuronal circuitry manifests as neurocognitive problems [44]. In this regard, high levels of HIV-1 Env (5 nM) have been shown to induce cytoskeletal changes in neurons [45]. Gp120 transgenic mice also exhibit decreased dendrites and synapses in addition to reactive gliosis in the brain [8,46]. The neuropathological findings were reflected in altered behavior on learning and memory tasks [46,47]. Although antiretroviral therapy has greatly extended the normal lifespan of people living with HIV infection, neurocognitive impairment in the setting of aging and inflammation remains an urgent problem [48,49]. Therefore, it is imperative to evaluate the changes that take place in the CNS microenvironment during HIV infection and to assess the collective contributions from viral particles and the ensuing inflammatory signaling.

In this report, we studied the role of Opn, a secreted protein elevated during neuroinflammation in the HIV infected brain, in modulating hippocampal synaptic architecture [13,15]. Opn is a known biomarker for various pathologies both in the CNS and periphery with circulating serum levels found to be >100 ng/mL [20,21,37,38]. Opn’s function in the brain has been regarded as both protective and detrimental, depending on the cellular location, pathology, and post-translational modification [16,17,18]. Opn expression has been previously shown to be elevated in developing neurons, implicating its role in the differentiation and maturation of neurons in the developing brain [50,51]. It is multifunctional, having pro-angiogenic, pro-survival, chemotactic, as well as adhesive properties. Opn can be post-translationally modified by phosphorylation, glycosylation, and by proteolytic cleavage by thrombin or matrix metalloproteinases [29,52,53]. Opn as a secreted protein can induce an outside-in stimulant for integrins by directly binding to them [54,55].

Due to its diverse features, its role in several neurological disorders including neuroHIV remains under-explored. Hence, we decided to investigate the role of Opn at serum level concentration of 100 ng/mL on neurons to understand its effect at the molecular level in the presence of HIV-1 Env. In contrast to other reports [45], we utilized a low concentration of HIV Env (400 pM) to also match the serum levels found in patients (100–800 pM) [56]. Additionally, we chose hippocampal neurons in our experiments due to their key function in the brain in learning and memory. Moreover, the hippocampus is one of the key regions in the brain that is negatively impacted by HIV [46]. Synaptic degradation in the hippocampus is apparent in HIV infection and the pathways are not completely understood [46,57]. The degradation is reportedly a combinatory effect by soluble factors released by the virus and circulating pro-inflammatory cytokines [7].

We showed here that elevated levels of extracellular Opn in the presence of HIV-1 Env can counteract the detrimental effect of HIV-1 Env on post-synaptic PSD-95 in primary rat hippocampal neurons. The presence of HIV-1 Env reduced the expression of PSD-95 and decreased its translocation to the spines thereby reducing synapse density. A decline in the number of post-synaptic spines indicates a decrease in the number of contacts being formed with the pre-synaptic membrane and hence, would result in the weakening of excitatory connections [10]. Adding Opn as a co-treatment recovered their number comparable to vehicle treated neurons, and distributed them to the spines according to our experiments.

We targeted *β*1 and *β*3 integrins to determine whether Opn acts on these receptors to exert a protective effect in the presence of HIV-1 Env. In that regard, if Opn utilizes any of these integrins then the number of dendritic spines would be expected to decrease with a reduction of integrin expression. However, silencing *β*1 integrins did not reduce, but rather increased the number of spines in the presence of Opn and HIV-1 Env. Therefore, we believe these integrins are involved in spine regulation in the co-presence of Opn and HIV-1 Env. *β*1 integrins have been previously shown to be highly involved in neuronal plasticity and can increase or decrease excitability depending on the ligand [25,26]. The proteolytic enzyme MMP9 is usually released by neurons due to increased activity [24]. This enzyme can then digest the ECM including PNNs and release soluble cell adhesion molecules (CAMs) [24,58]. One of these CAMs is the intercellular adhesion molecule-5 (iCAM), which was shown to stimulate *β*1 integrins [58,59,60]. This activation of integrins promoted dendritic spine enlargement via cofilin inactivation and F-actin polymerization in hippocampal slices [25,35]. On the other hand, in the presence of the astrocytic derived extracellular matrix protein thrombospondin, *β*1 integrins depolymerized F-actin and lead to endocytosis of synaptic receptors in cultured spinal cord neurons thereby reducing neuronal excitability [26]. From our results, we noted a decrease in the PNN/*β*3-tubulin expression in *β*1 integrin silenced neurons co-treated with Opn and HIV-1 Env. Degradation of PNNs have been suggested to promote dendritic spine formation [24] which could explain why we observed an increase in spine density in *β*1 integrin silenced neurons co-treated with Opn and HIV-1 Env.

We also observed similar decrease in PNN/*β*III-tubulin expression and an increase in spine density in Opn only treated neurons. Opn has been shown to increase the expression of MMP9 via *β*3 integrins in Neuro2A cells, human chondrosarcoma cells, cardiac, skeletal muscles, and endometrial cells [22,61,62,63]. It is possible that they also increase MMP9 in this context and promote spine density by decreasing PNN expression and that needs to be shown in future studies. The increase in spine density was reversed by silencing *β*3 integrins suggesting these to be targeted by Opn to modulate dendritic spines. However, *β*3 integrins did not modulate the expression of PNNs as we did not see any difference in PNN levels when they were silenced. *β*3 integrins have been previously found to regulate synaptic maturation by enhancing the probability of neurotransmitter release and increasing post-synaptic density in developing hippocampal neurons [64]. *β*3 integrins have also been shown to mediate hippocampal dendritic spine remodeling via actin reorganization through calcium dependent mechanisms [33]. Therefore, *β*3 integrins in the presence of Opn are believed to positively enhance the number and density of post-synaptic structures without regulating the expression of PNNs. In the absence of these integrins, HIV-1 Env-only treated neurons exhibited even lower spine density suggesting the dysregulation prompted by HIV-1 Env is exacerbated by knocking the expression of *β*3 integrins down. This alludes to an intrinsic function of *β*3 integrins in the presence of HIV-1 Env and adds to the evidence that integrins themselves may have roles in mediating synaptic plasticity in the presence of HIV-1 Env. However, we did not observe this protective effect of *β*3 integrins in the co-presence of Opn and HIV-1 Env, suggesting a complex cross-talk between the three. Future studies involving the detailed interaction between HIV-1 Env and *β*3 integrins are therefore warranted. Further investigations into the intracellular signals regulated by *β*3 integrins might also shed light on whether there is a feedback loop due to the presence of both Opn and HIV-1 Env.

In conclusion, our work demonstrates post-synaptic modulation of hippocampal neurons by HIV-1 Env, Opn and integrins and predicts some of the mechanism by which the synaptic integrity can be impaired during HIV infection. We show here that pathological levels HIV-1 Env can reduce the expression of PSD-95 and reduce the number of dendritic spines. This would result in a reduced number of contacts being formed with the post-synaptic machinery and thus suggests weaker synaptic transmission. The ECM acts to counteract this damage by allowing the neurons to be more plastic. The ECM is very complex and we have only studied a few of the components here but offers an exciting avenue for further exploration. This includes but is not limited to the role of MMP9s and the dynamics of PNNs in the presence of HIV-1 proteins. Future studies using specific PNN markers are therefore warranted to understand the complexity that arises in the presence of HIV-1 Env. Their composition changes, if any, might give a clue as to how synaptic function is dysregulated.

In addition, we have shown here how secreted phosphoprotein Opn exerts a protective effect on synapses. However, there are some limitations as our studies were performed in in vitro cultures and do not completely capture the complexity of the brain. Therefore, future in vivo or ex vivo studies with Opn knockout mice and gene transfected organoid cultures are warranted to confirm whether our in vitro findings are reflected as molecular changes in the brain, and associated with the expected behavioral changes perhaps recapitulating aspects of deficits in fact observed during neuroHIV.
